# Serum thyroglobulin levels are predictive of urinary iodine concentration thresholds for defining population iodine status

**DOI:** 10.1017/S0007114525105205

**Published:** 2025-11-14

**Authors:** Kasthuri Sivalogan, Rafael Flores-Ayala, Roelinda Jongstra, Carolina Martinez, Roberto Mendoza, Mireya Palmieri, Karla Mesarina, Maria Elena Jefferds, O. Yaw Addo

**Affiliations:** 1 Nutrition and Health Sciences Doctoral Program, Emory University, Atlanta, GA, USA; 2 Nutrition Branch, USA Centers for Disease Control and Prevention (CDC), Atlanta, GA, USA; 3 McKing Consulting Corporation, Atlanta, GA, USA; 4 Institute of Nutrition of Central America and Panama (INCAP-Guatemala), Guatemala City, Guatemala; 5 Secretariat of Food and Nutrition, Guatemala City, Guatemala

**Keywords:** Iodine, Thyroglobulin, Median urinary iodine, Biomarker assessment

## Abstract

Thyroglobulin (Tg) has been considered a measure of iodine status, but there is no global guidance. This analysis examines the relationship between serum Tg and spot urinary iodine concentration (UIC) data to identify Tg concentrations that correspond to current WHO thresholds for population iodine status. We analysed data from 730 non-pregnant Guatemalan women aged 15–49 years who had both UIC and Tg measurements. Correlations were examined. Bootstrap stratified finite sampling with replacement was used to generate cluster *k-medians* of UIC (mUIC) and Tg (mTg) that served as the population unit of analyses. Non-linear restricted cubic spline regression dose–response curve functions and ordinary differential equations were then used to derive the Tg threshold corresponding to WHO definitions for UIC. Mean age was 30·2 (sd 9·3) years. mTg was 10·4 ng/ml (9·9, 10·8), and mUIC was 148·7 μg/l (139·1, 161·0). Correlations between spot UIC and Tg were NS at the individual level, but correlations based on population *k-medians* were significant (Spearman *r* = −0·21 to −0·06, each *P* < 0·0001) and demonstrated a *U*-shaped relationship according to WHO categories. Derived mTg cutoffs were 14·2 ng/ml predictive of UIC insufficiency, 10·2 ng/ml for UIC adequacy, 8·5 ng/ml for UIC above adequate and 10·8 ng/ml for UIC excess. The significant and graded mUIC–mTg correlations suggest that Tg concentrations predictive of UIC categories are obtainable for non-pregnant Guatemalan women aged 15–49 years. The newly derived mTg cutoff may be more discriminant at a lower spectrum of UIC in terms of identifying iodine-deficient women, more so than in the UIC excess category.

Iodine is an exogenous essential micronutrient and a necessary component of thyroid hormones. As a result, iodine deficiency disorders are one of the most common micronutrient deficiencies at all life stages, with potentially irreversible consequences during pregnancy and for fetal and newborn health. While global recommendations for universal salt iodisation and fortification have contributed to reductions in iodine deficiency and iodine deficiency disorder, 35–45 % of the world’s population is still at risk^(1)^. Iodine insufficiency may impair thyroid hormone production, leading to iodine deficiency disorders such as goitre, hypothyroidism and cognitive impairments, while excess iodine intake can also disrupt thyroid function, potentially resulting in hyperthyroidism or autoimmune thyroiditis^(2)^.

The WHO currently recommends urinary iodine concentration (UIC) as the gold standard marker of recent dietary iodine intake^(3)^. Spot UIC is a highly variable measure of recent intake and is used to assess population iodine status, while it cannot be used to assess individual intake because of large intra- and interindividual variation^(4)^. While iodine content in a 24 h urine sample may be more reliable, it is associated with high respondent burden and is often not feasible in population surveys^(5)^. As a result, general practice is to use median population levels from spot urine collections, and median UIC (mUIC) cutoff ranges exist for pregnant and non-pregnant women populations 15–49 years of age^(2,6)^.

Thyroglobulin (Tg) is a scaffold protein in which thyroid hormones are synthesised and can be a measure of iodine status in all populations and among those with iodine deficiency and excess intake^(7–10)^. Small amounts of Tg are released into circulation in iodine sufficiency and exhibit a *U*-shaped relationship with iodine intake. Tg levels increase in iodine deficiency due to thyroid-stimulating hormone hyperstimulation and thyroid hyperplasia^(4)^. Excess iodine can inhibit Tg proteolysis, but persistent intake can then increase Tg since the thyroid gland fails to escape from the Wolff–Chaikoff effect^(4)^. As a result, studies have indicated that Tg can be considered a biomarker for population iodine status and is a useful measure in iodine deficiency and excess^(11)^.

Median Tg (mTg) levels for iodine sufficiency were initially reported by the WHO but removed due to lack of or limited evidence^(9,12)^. Currently, Tg concentrations associated with iodine status only exist for school-aged children because the current WHO biomarker guidance was developed with the premise of capturing whether a population group of school-age children is iodine-deficient or not^(13–15)^. However, there are no established Tg concentrations that correspond to published WHO mUIC thresholds for population iodine status, including deficiency and excess, which are necessary for population-level iodine status monitoring.

At a global scale, twenty-one countries had insufficient iodine intake in 2020 based on national or subnational surveys among women of reproductive age, school-aged children, adolescents and adults^(14)^. The Sistema de Vigilancia Epidemiológica de Salud y Nutrición (Epidemiological Health and Nutrition Surveillance System, SIVESNU) data indicate that 79·6 and 22·1 % of women reported consuming foods prepared with coarse and refined salt, respectively, the day before the survey in 2017. In addition, mUIC was 145 μg/l among non-pregnant women, indicating population-level adequacy^(16)^. Moreover, in assessing a country’s iodine status, it is recommended that school-age children be used as a proxy for the general population, but there are concerns that the national data in Guatemala may not reflect subnational differences or iodine status among specific subgroups of interest, such as women aged 15–49 years^(17,18)^.

The objective of this analysis is to examine Tg levels that correspond to the current WHO published thresholds for mUIC population iodine status, using national data from non-pregnant Guatemalan women aged 15–49 years.

## Study methods

### Data source

Data from this study came from the SIVESNU surveillance system, which is a complex design, nationally representative, cross-sectional, continuous household survey conducted approximately annually in Guatemala since 2013. SIVESNU uses a multistage sampling approach to capture data on several health and nutrition topics, including UIC, and in 2018/2019, it also assessed Tg, among women aged 15–49 years. Detailed information on the SIVESNU surveillance system, study area, study population and sampling strategy can be found elsewhere^(19)^. In 2018–2019, thirty households were randomly selected from each of the 100 eligible clusters, and one non-pregnant woman aged 15–49 years from each household was randomly selected to participate. There was no replacement of clusters, households or selected individuals within the households for any reason^(19)^. Questionnaire, anthropometry data, blood and urine biospecimen collections occurred at the same visit for the majority of the women sampled. In isolated cases where only questionnaire data were collected, enumerators returned to the household for blood and urine sample collection at a later date, usually within 2 d of working in each cluster.

### Questionnaire data collection

Data were collected using a household questionnaire and women aged 15–49 years. Trained enumerators administered the surveys in Spanish or in a local indigenous language, via an interpreter, using validated instruments. The women’s survey included questions about health status, physical activity, reproductive history and dietary diversity, among other topics. Further information on data collection for the 2018–2019 survey cycles can be found online at the Informe del Sistema de Vigilancia Epidemiológica de Salud y Nutrición (SIVESNU) (https://www.siinsan.gob.gt/siinsan/monitoreo-y-evaluacion/#).

### Informed consent

The Guatemalan Ministry of Health Institutional Review Board approved the 2018–2019 SIVESNU cycle. Adults provided written informed consent prior to participating in the survey. A de-identified dataset was used for analytical purposes.

### Blood sampling

Venous blood samples (approximately 500 μl) were collected to assess serum Tg (ng/ml) using electrochemiluminescence immunoassay, Cobas E411 analyser (Roche Diagnostics International Ltd). Measurement range for serum Tg was 0·04–500 ng/ml, with a limit of detection of 0·04 ng/ml. After field collection in 2017–18, the residual serum specimen was stored at −70°C until funds became available for laboratory analyses in May 2021.

### Urine sampling

Urine was collected from women to assess UIC. Each woman was given a sterile wide-mouth cup to provide a small amount of urine (approximately 6 ml). Specimens were transferred to plastic tubes and stored in the field in cold boxes within clusters and later transported to the Institute of Nutrition of Central America and Panama (Instituto de Nutrición de Centroamérica y Panamá) laboratory in Guatemala City and refrigerated at 2–8°C until they were processed using the standard ammonium persulfate method^(20)^. After collection, urine specimens were transported to the Institute of Nutrition of Central America and Panama (Instituto de Nutrición de Centroamérica y Panamá) at the end of each round (3 weeks of field work) in the 2017–2018 survey cycle, with laboratory processing occurring at a later subject to the availability of funds.

### Statistical methods

The analysis was restricted to non-pregnant women aged 15–49 years with both Tg and UIC measurements. All analyses were conducted in SAS (SAS Institute) and R (R Foundation for Statistical Computing). Descriptive statistics were presented as unweighted means, medians and percentages, and mTg and UIC were calculated by participant characteristics. Bivariate analyses and visual median scatter plot analyses were used to examine the monotonic relationships between Tg and UIC. Spearman correlations between Tg and UIC at the individual and population cluster levels were used to assess the strength of bivariate relations.

### Analytic construct

To address challenges with the wide variability of urinary iodine concentration and Tg levels, we used a statistical approach that is based on the median, the most stable statistic of the analyte distribution^(21)^.

We adopted methods in combinatorics to generate population sub-clusters, and permutation sampling to calculate population *k-cluster medians* of UIC and Tg that correspond to the WHO definition’s for mUIC (insufficiency (<100 μg/l), adequacy (100–199 μg/l), above adequate (200–299 μg/l) and excess (>300 μg/l))^(3,22–24)^. This *k*-medians approach facilitates population-cluster analyses of Tg and UIC, rather than extrapolation at the individual level, and aligns with current WHO iodine guidance^(2)^. In addition, *K*-median (cluster median) is robust to outlaying concentrations, so limits of detection or out-of-range values were not of concern and not excluded. Bootstrap sampling was used to generate finite population stratified unrestricted random samples from the original data of Guatemalan women^(24)^. The analytic inclusion criteria were women with non-missing age, UIC and Tg values. Age of the women was categorised into 15–19, 20–29, 30–40 and 40–49 years, while mUIC was categorised into the four WHO cutoffs, which resulted in 4 × 4^(16)^ combinations; each had equal probabilities, with no specific order of selection within the sixteen finite and independent occurrences. These two classifier variables served as the strata for our stratified unrestricted random samples with replacement, which ensured that each population cluster would have women of age and UIC groupings (and resemble the original population). A total of 500 bootstrap replicates were used. The *K*-median estimates of UIC by each finite Tg cluster can be summarised as 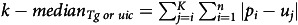 , where *k* is the number of population clusters, *u*_*j*_ is the median vector of the cluster *j* and *p* is a vector of instances in the dataset with sample size *n* ^(25)^.

#### Concentration dose–response curve analysis

To identify Tg values predictive of WHO mUIC categories, non-parametric restricted cubic spline modelling, using the *k-medians* as the unit of analysis, a mUiC and mTg concentration curve function was fit. Ordinary differential equations were then applied to each mUIC–mTg curve function to identify the physiological inflection point value of Tg that is predictive of mUIC inadequacy, adequacy, above adequate and excess. The inflection points represent the curve minimum, denoting an instantaneous change in slope as 2nd-order derivative (*ΔmUIC* ^*2*^ */ΔmTg* ^*2*^ *)* function of mTg. Derived Tg cutoff point estimates and 95 % CI were calculated with bootstrap replication.

## Results

Data used in the analyses came from the ninety-six (out of 100) eligible clusters that accepted to participate in the 2018–2019 SIVESNU cycle. A total of 2880 households were identified in the ninety-six enumeration areas, with 2424 households agreeing to participate. Among the 1989 women (both pregnant and non-pregnant) aged 15–49 years who were identified, 1755 women agreed to participate, yielding a response rate of 87·5 %. Of those, 1573 women provided urine samples for UIC assessment, while about half (845 women) provided blood samples for Tg measurement. Our final sample size for this analysis was *n* 730 non-pregnant women aged 15–49 years with both Tg and UIC measurements. However, our study population did include 116 lactating non-pregnant women.

Characteristics of this study population are presented in Table 1. Mean age (sd) was 30·2 (sd 9·3) years. mTg (95 % CI) was 10·37 ng/ml (9·93, 10·84), and mUIC (95 % CI) was 148·72 μg/l (139·09, 161·01) (data not shown). The distribution of mTg and mUIC is presented in Tables 2 and 3 for non-pregnant women aged 15–49 years by sociodemographic characteristics. Women who only provided urine samples and were not included in the analysis (*n* 942) were comparable to women with both Tg and UIC data available who were included in the analysis (*n* 730) in terms of mean age, ethnicity, type of residence and socio-economic status.

**Table 1.** Descriptive statistics of study population for non-pregnant women aged 15–49 years, Epidemiological Health and Nutrition Surveillance System, 2018–2019, Guatemala

	Percentage	Sample size/*n*
Age groups (years)		
15–19	13·40 %	98
20–29	34·90 %	255
30–39	31·60 %	231
40–49	19·90 %	145
Residence		
Urban	63·20 %	461
Rural	36·80 %	269
Ethnic groups		
Indigenous	39·00 %	285
Non-indigenous	64·90 %	445
Multidimensional Poverty Index		
Not severe	80·30 %	586
Severe	19·70 %	144
Household income status		
Low	34·20 %	250
Middle	34·50 %	252
High	31·10 %	227

**Table 2.** Distribution of median (IQR) thyroglobulin (mTg) and urinary iodine concentrations (mUIC) by sociodemographic characteristics for non-pregnant women aged 15–49 years, Epidemiological Health and Nutrition Surveillance System, 2018–2019, Guatemala

	mTg	IQR	*n*	mUIC	IQR	*n*
Age (years)						
15–19	9·20	6·16–12·33	98	145·89	88·35–261·55	98
20–29	11·51	7·57–16·93	255	150·95	73·69–250·50	255
30–39	9·79	6·04–5·89	231	148·79	76·34–244·89	231
> 40	11·95	7·80–17·17	145	148·67	77·82–259·21	145
Residence						
Urban	10·53	6·49–15·54	461	190·85	112·08–275·37	461
Rural	10·70	7·03–16·59	269	126·20	63·83–219·10	269
Ethnic group						
Indigenous	10·79	7·54–16·61	285	103·72	52·48–193·06	285
Non-indigenous	10·48	6·28–15·94	445	179·29	102·35–281·40	445
Multidimensional Poverty Index						
Not severe	10·58	6·59–16·30	586	153·42	81·35–263·07	586
Severe	10·75	7·40–16·72	144	125·11	62·18–211·04	144
Household income status						
Low	10·84	7·12–16·53	250	142·34	72·72–250·01	250
Middle	10·26	6·52–16·05	252	137·33	70·93–234·15	252
High	10·73	6·56–16·61	227	178·62	97·99–246·79	227

**Table 3.** Correlations (*r*, *P*-value) between thyroglobulin and WHO median urinary iodine concentration (mUIC) categories among non-pregnant women aged 15–49 years, Epidemiological Health and Nutrition Surveillance System, 2018–2019, Guatemala

	WHO mUIC categories							
	Insufficient UIC (< 100 μg/l)		Adequate UIC (100–199 μg/l)		Above adequate UIC (200–299 μg/l)		Excessive UIC (> 300 μg/l)	
Individual correlations	0·00	0·99	–0·08	0·24	–0·06	0·46	–0·10	0·26
Population *K*-median correlations	–0·22^*^		–0·12^*^		–0·10^*^		–0·12^*^	

* Significant at *P* < 0·0001.

**Fig. 1.**
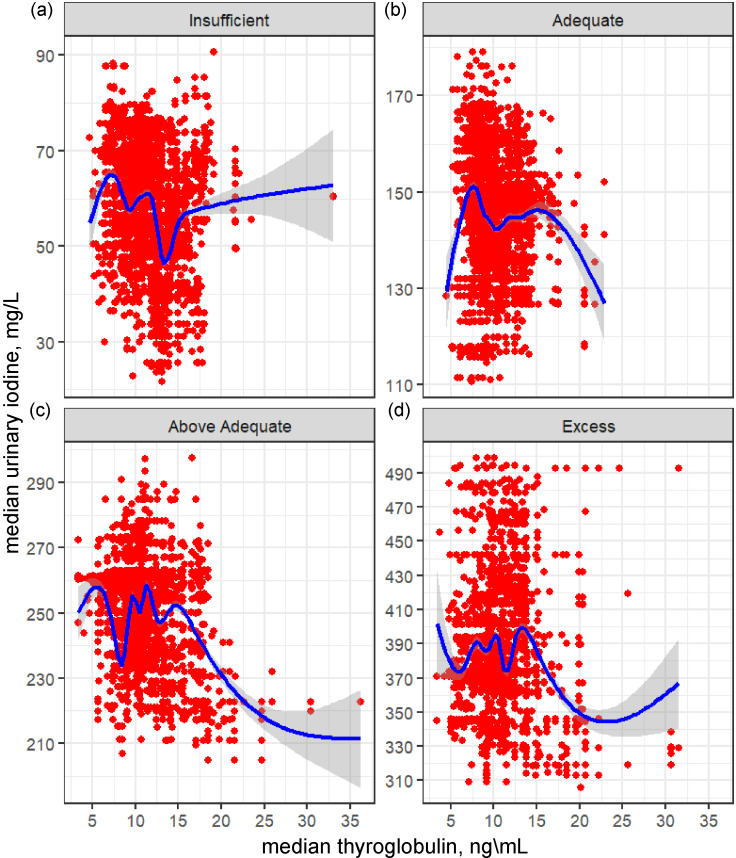
Monotonic trends for median thyroglobulin (mTg) and median urinary iodine concentrations (UIC) by WHO population median UIC categories among non-pregnant women aged 15–49 years, Epidemiological Health and Nutrition Surveillance System, 2018–2019, Guatemala. ^1^Clockwise from top left: (a) UIC insufficient population; (b) UIC adequate population; (c) UIC above adequate population; (d) UIC excess population.

Figure 1 used exploratory data analysis of a scattergram and a median curve overlay to visualise monotonic trends for mTg and mUIC by WHO population mUIC categories^(26)^. The visual display indicated that within each mUIC category, a non-linear dose–response relationship exists between Tg and mUIC and is characterised by inflections and points of stabilisation. Because exploratory data analysis lack a closed-form equation, a statistical method that fits a curve function was applied to further explore Tg concentration associated with the inflection points. Table 4 presents the individual-level and population-level *K-medians* correlation between Tg and UIC for all WHO mUIC categories. Correlations were NS at the individual level but were significant (*P* < 0·0001) at the population level.

**Table 4.** Predictive median thyroglobulin (mTg) corresponding to WHO median urinary iodine concentration (mUIC) population categories among non-pregnant women aged 15–49 years, Epidemiological Health and Nutrition Surveillance System, 2018–2019, Guatemala

		mTg, ng/ml	
		Threshold	95 % CI
mUIC	Insufficient UIC (< 100 μg/l)	14·05	14·01, 14·09
	Adequate UIC (100–199 μg/l)	9·83	9·79, 9·88
	Above adequate UIC (200–299 μg/l)	8·55	8·53, 8·57
	Excess UIC (> 300 μg/l)	11·40	11·38, 11·43

**Fig. 2.**
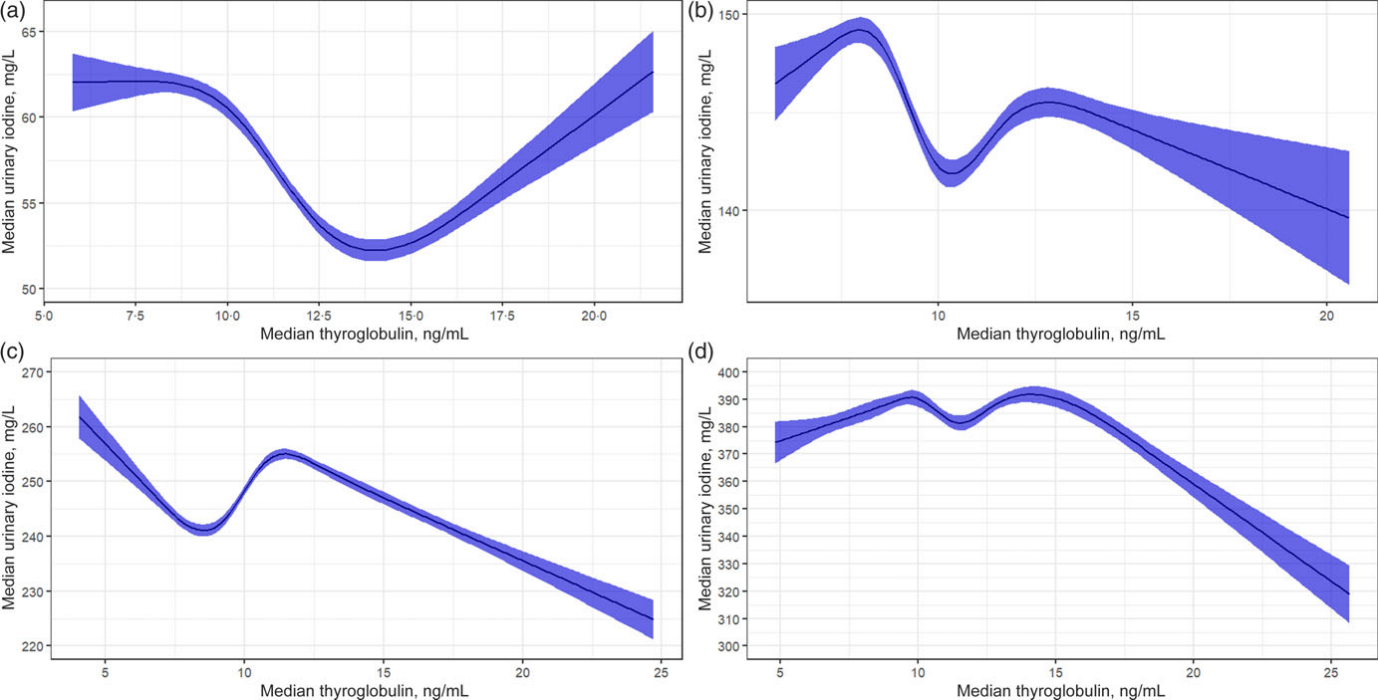
Restricted cubic spline curve for median urinary iodine concentration categories for insufficient (a), adequate (b), above adequate (c) and excess (d) subpopulations, among non-pregnant women aged 15–49 years, Epidemiological Health and Nutrition Surveillance System, 2018–2019, Guatemala.

Results from the restricted cubic spline non-parametric curve function analysis are presented in Fig. 2. mTg values predictive of mUIC insufficiency, adequacy, above adequate and excess are reported in Table 4, along with 95 % CI. mTg predictive of mUIC categories are consistent with the *U*-shaped biological relationship between Tg and UIC. mTg values were 14·1 ng/ml for mUIC insufficiency, 9·8 ng/ml for mUIC adequacy, 8·6 ng/ml for mUIC above adequate and 11·4 ng/ml for mUIC excess.

## Discussion

This novel analysis uses innovative statistical methods to demonstrate that Tg is a functional biomarker of population iodine status and is significantly correlated with UIC at different mUIC thresholds at the population level. We were able to generate mTg values that are predictive of and correspond to WHO mUIC population categories for insufficiency, adequacy, above adequate and excess among non-pregnant Guatemalan women aged 15–49 years. In addition, these results indicate that physiological mTg thresholds are feasible and can capture the biological relationship with mUIC at different spot urinary iodine levels. mTg values were highest corresponding to mUIC insufficiency (14·1 ng/ml), decreased corresponding with mUIC adequacy (9·8 ng/ml) and above adequate (8·6 ng/ml) values and increased again when corresponding to mUIC excess (11·4 ng/ml) values. The mTg values demonstrated a *U*-shaped relationship with mUIC categories consistent with the literature and our understanding of Tg and iodine biology^(10)^. Further, mTg-mUIC correlations were strongest in the mUIC insufficient (*r* = –0·22, *P* < 0·0001) category, suggesting that the newly derived mTg cutoff may be more discriminant in terms of identifying iodine insufficient women, more so than those in the UIC excess categories that had lower explained variance relative to insufficient status. As Tg is a homeostatically controlled protein, it gets expressed in both iodine-deficient and excess states^(27,28)^. Therefore, further examination of other thyroid-related biomarkers, which were unavailable in this Guatemala database, such as triiodothyronine, thyroxine, thyroid-stimulating hormone and triiodothyronine:thyroxine ratio using similar analytical methods, may also facilitate identifying individuals with excess iodine. And finally, consumption of iodised salt foods was at 74 %, and mean iodine in household samples was 21·0 (17·2) mg/kg with a range of 0·0–120·2 mg/kg. This evidence provides support that Tg levels identified in this analysis are likely to be minimally impacted by a lack of iodised salt in the food environment^(29)^.

Although current global recommendations endorse UIC measurement from single or spot urine samples, a physiologically based biomarker for iodine status such as Tg may be more appropriate as it is a more stable and integrative indicator of iodine status, especially in women aged 15–49 years who may have fluctuating intake or increased physiological demands. However, currently, iodine studies with Tg measurement in the literature are challenging to interpret for individual-level application and population-level assessment^(30–32)^. First, Tg is not sensitive to recent changes in iodine intake^(31)^. Second, studies investigating functional biomarkers of iodine status in the literature have used different outcomes and less robust analytical methods^(32)^. Additionally, comparisons between Tg values in the published literature are challenging to interpret due to differences in assay methods and cut points, which can confound comparisons across studies^(31)^. And finally, studies examining Tg and UIC among adults, or within specific subgroups such as women aged 15–49 years or pregnant women, tend to generate one mTg and one mUIC value for the population of interest, instead of values corresponding to the different WHO thresholds^(4)^. For example, a systematic review of twelve observational studies in adults suggests that Tg concentrations < 13 or ≥ 13 µg/l are not an appropriate cutoff for identifying iodine-sufficient and iodine-deficient populations of adults. This was due to the fact that eight of the twelve studies reported that iodine-deficient adults had a mTg < 13 µg/l, which is quite consistent with the 14·2 ng/ml mTg threshold identified in our study^(4)^. The 13 µg/l threshold was derived from an earlier multicentre study conducted among school-aged children, which concluded that mTg < 13 µg/l and/or < 3 % of Tg values > 40 µg/l could be used as a biomarker of adequate iodine status in children^(10)^.

Two studies by Shi *et al.* (2015) and Stinca *et al.* (2017) further corroborate that mTg ∼10 µg/l can be used to categorise iodine sufficiency in pregnant and non-pregnant women. Shi *et al.* (2015) derived a 10 µg/l cutoff by assessing serum Tg concentrations across different UIC concentrations, while Stinca *et al.* (2017) derived their cutoff based on the median dried blood spot-Tg and 95 % CI from a reference pregnant women population with an adequate mUIC^(33,34)^. Our approach is based on the normative biological relationship between Tg and iodine excretion. These results suggest that harmonisation of Tg cutoffs for assessing iodine status in population surveys may be warranted in the future^(35–38)^.

Examining additional data on Tg and UIC measures that replicate this analysis in different population groups – particularly to compare results from pregnant and non-pregnant women with varying iodine intakes and in populations with and without iodine salt fortification – may be useful to help harmonise thresholds. Universal salt iodisation mandates and the inclusion of iodised salt in processed foods have resulted in increased salt fortification and iodine intake; over-consumption of iodine has negative health implications such as hyperthyroidism or hypothyroidism^(38,39)^. Thus, reliable and accessible iodine monitoring is necessary, especially given the co-occurrence of iodine deficiency and iodine excess in some populations^(40)^.

There are many strengths in this analysis. This analysis was conducted using a large sample size of Guatemalan women and a wide range of UIC. In addition, the results of this analysis are a step towards micronutrient surveillance and can be used as further evidence for the use of Tg as a biomarker for iodine assessment and monitoring. Furthermore, UIC is not a clinical measure and is not available in electronic health records, but thyroid panels are routinely ordered in many settings, so it may be possible to use Tg data from electronic health records for population surveillance of iodine status. However, there is a limitation that needs to be considered in the interpretation of the results. This analysis was conducted using nationally representative survey data from non-pregnant women aged 15–49 years in Guatemala and may not be representative of other populations, including pregnant women, children, adolescents and the elderly, populations with different salt iodisation policies and/or dietary salt intake. In addition, since data came from household nutrition surveys, we were unable to examine a full immunology panel as related to Tg, as would be the case in a clinical setting.

### Conclusion

In this sample of Guatemalan non-pregnant women aged 15–49 years, there was significant and graded correlations between mUIC and mTg, supporting that Tg concentrations are predictive of WHO mUIC categories. Additional validation, harmonisation of cut points and assay methods would be necessary to determine if Tg could be a potentially viable biomarker for assessing population iodine status in women.
